# Black–White and Country of Birth Disparities in Retention in HIV Care and Viral Suppression among Latinos with HIV in Florida, 2015

**DOI:** 10.3390/ijerph14020120

**Published:** 2017-01-27

**Authors:** Diana M. Sheehan, Daniel E. Mauck, Kristopher P. Fennie, Elena A. Cyrus, Lorene M. Maddox, Spencer Lieb, Mary Jo Trepka

**Affiliations:** 1 Department of Epidemiology, Robert Stempel College of Public Health and Social Work, Florida International University, 11200 SW 8th St, Miami, FL 33199, USA; dmauck@fiu.edu (D.E.M.); kfennie@fiu.edu (K.P.F.); 2 Center for Substance Use and HIV/AIDS Research on Latinos in the United States (C–SALUD), Florida International University, 11200 SW 8th St, Miami, FL 33199, USA; ecyrusca@fiu.edu; 3 HIV/AIDS Section, Florida Department of Health, 4052 Bald Cypress Way, Tallahassee, FL 32399, USA; lorene.maddox@flhealth.gov; 4 Florida Consortium for HIV/AIDS Research/The AIDS Institute, 410 Victory Garden Dr., Suite 127, Tallahassee, FL 32301, USA; slieb@comcast.net; 5 Department of Epidemiology and Center for Substance Use and HIV/AIDS Research on Latinos in the United States (C–SALUD), Robert Stempel College of Public Health and Social Work, Florida International University, 11200 SW 8th St, Miami, FL 33199, USA; trepkam@fiu.edu

**Keywords:** Latinos, racial disparities, birth country disparities, human immunodeficiency virus, retention in HIV care, viral suppression

## Abstract

The study’s purpose was to identify HIV, Black–White race, and birth country disparities in retention in HIV care and HIV viral load (VL) suppression among Latinos, in 2015. Florida’s surveillance data for Latinos diagnosed with HIV (2000–2014) were merged with American Community Survey data. Multi-level (random effects) models were used to estimate adjusted odds ratios (aOR) for non-retention in care and non-viral load suppression. Blacks and Whites experienced similar odds of non–retention in care. Racial differences in VL suppression disappeared after controlling for neighborhood factors. Compared to U.S.–born Latinos, those born in Mexico (retention aOR 2.00, 95% CI 1.70–2.36; VL 1.85, 95% CI 1.57–2.17) and Central America (retention aOR 1.33, 95% CI 1.16–1.53; VL 1.28, 95% CI 1.12–2.47) were at an increased risk after controlling for individual and neighborhood factors. Among Central Americans, those born in Guatemala (retention aOR 2.39, 95% CI 1.80–3.18; VL 2.20, 95% CI 1.66–2.92) and Honduras (retention aOR 1.39, 95% CI 1.13–1.72; VL 1.42, 95% CI 1.16–1.74) experienced the largest disparities, when compared to U.S.-born Latinos. Disparities in care and treatment exist within the Latino population. Cultural and other factors, unique to Latino Black-White racial and birth country subgroups, should be further studied and considered for intervention.

## 1. Introduction

Both the Joint United Nations Programme on HIV/AIDS and the United States (U.S.) National HIV/AIDS Strategy, call for significant improvements in retention in HIV care and viral load suppression among individuals with HIV, in order to end the national and worldwide HIV/AIDS epidemic [1,2]. Poor retention in HIV care has been associated with delayed viral load suppression [3] and increased risk of mortality [4]. Low-level viremia has been associated with improved immunologic response [5], delayed time to opportunistic infection [6], and decreased risk of sexual HIV transmission [7].

In addition, the National HIV/AIDS Strategy aims to reduce HIV-related disparities and health inequities [2]. A recent Centers for Disease Control and Prevention (CDC) study of 11 states and the District of Columbia, suggests that the proportion of Hispanics/Latinos with HIV who are consistently retained in care (49.5%) is higher than for non-Latino Whites (48.6%) and non-Latino Blacks (37.7%) [8]. Furthermore, the proportion of Latinos in the US in care who are virally suppressed, is reportedly similar to that of non-Latino Whites (31% vs. 32%, *p*-value 0.91). However, previous studies by our group and others have shown significant disparities within racial and birth country subgroups of Latinos when focusing on HIV risk [9], delayed HIV diagnosis [10,11,12], and survival after HIV [10,13,14,15]. Only one study to date has examined retention in care among Latinos by country of birth (or country of origin), but this research simply reported the findings for Latinos born in Mexico and Central America, and small sample sizes precluded multivariate modeling or in-depth analysis [16]. To our knowledge, no studies have examined viral suppression by country of birth, or retention in care and viral suppression by race, among Latinos. 

Furthermore, Latinos differ in socioeconomic status, with Black Latinos, and Mexican and Central American Latinos, more likely to experience poverty, lower educational attainment, and unemployment, than their White and U.S.-born counterparts [17,18]. These differences in individual-level socioeconomic status likely create disparities in exposure to neighborhood disadvantage. Low neighborhood socioeconomic status and neighborhood poverty have been associated with high HIV rates [19,20], and low AIDS survival [21,22], and have been shown to partially account for racial/ethnic disparities in HIV/AIDS survival [23,24], and antiretroviral initiation [24].

Given the vital individual and public health benefits of retention in care and viral suppression, and the wide health and socioeconomic disparities previously reported, it is important to assess whether differences in retention in care and viral suppression exist within Latino racial and birth country subgroups, in order to identify areas for targeting interventions. Therefore, the objective of this study was to identify potential disparities by race and by country of birth in retention in HIV care and HIV viral load suppression, among Latinos with HIV. Furthermore, we sought to identify contributing individual- and neighborhood-level risk factors unique to each racial and country of birth subgroup. 

## 2. Data and Methods

### 2.1. Datasets

De-identified HIV surveillance records were obtained from the Florida Department of Health’s (FDOH) enhanced HIV/AIDS reporting system (eHARS). Cases reported as Latino or Hispanic (of any race), aged ≥ 13 years who met the Centers for Disease Control and Prevention (CDC) HIV case definition [25] during the years 2000–2014, listed Florida as the most current state of residence, and were alive at the end of 2015, were analyzed. Cases with missing or invalid data for the current ZIP code (n = 2866), a reported current ZIP code with a population of zero based on American Community Survey (ACS) estimates (n = 9), or those diagnosed in a correctional facility (n = 322), were excluded. Latinos born in an “unknown/unspecified” or missing country of birth (n = 1746), or in countries other than the U.S., Puerto Rico, Mexico, Cuba, Central America, and South America (n = 1001), were also excluded. No cases were missing data on retention in HIV care or viral suppression during 2015. The 2009–2013 American Community Survey (ACS) was used to obtain neighborhood-level data using ZIP code tabulation areas (ZCTAs) [26]. ZCTAs are used by the U.S. Census Bureau to approximate U.S. postal service ZIP codes for tabulating and reporting summary statistics [27]. 

### 2.2. Individual-Level Variables

Our primary individual-level variables of interest from eHARS were race and country of birth. These data were self-reported during HIV testing, reported by a health care provider, or extracted from medical chart reviews. Our study included only individuals reported as being of Latino/Hispanic ethnicity. Race was missing for 988 (8.2%) of the 12,106 Latinos who met our inclusion criteria. We imputed data for race using the PROC MI procedure in SAS, by creating 10 datasets. We used the year of HIV diagnosis, sex, age, country of birth, and HIV transmission mode, to predict race in a fully conditional specification (FCS) model [28]. All analyses were fitted by imputation, by constructing pooled estimates. Country of birth was missing for 1746 (9.3%) of all cases diagnosed between 2000 and 2014. Cases with a missing or unknown country of birth were excluded. Imputation was not conducted in this case, due to the many countries in which individuals could have been born and the limited individual-level data available for imputation. We grouped the country of birth into six categories: U.S., Puerto Rico, Mexico, Cuba, Central America, and South America. The U.S.-born category included individuals born in any of the 50 states, District of Columbia, or any U.S. dependent territory, but excluded Puerto Rico. Central America included Costa Rica, El Salvador, Guatemala, Honduras, Nicaragua, and Panama. South America included Argentina, Bolivia, Chile, Colombia, Ecuador, Paraguay, Peru, Uruguay, and Venezuela.

Our outcomes of interest were also extracted from eHARS and included retention in HIV care and HIV viral load suppression in 2015. Retention in care during 2015 was defined as evidence of engagement in care two or more times, at least three months apart, during 2015. Engagement in care was defined as evidence of at least one laboratory test, a prescription fill through the AIDS Drug Assistance Program (ADAP) (for those in ADAP), or a physician visit documented in one of the Ryan White databases (for those receiving services through the Ryan White HIV/AIDS program). HIV viral load suppression during 2015 was also defined and calculated by the FDOH as having evidence of a viral load <200 copies/mL in the last laboratory test, performed during 2015. The last viral load test during the measurement year is used by the U.S. Department of Health and Human Services’ HIV/AIDS Bureau to measure program performance [29]. Additionally, we extracted the following individual-level data from eHARS: HIV diagnosis year, sex at birth, age at HIV diagnosis, HIV transmission mode, AIDS diagnosis (if case progressed to AIDS by 31 December 2015), year of death, and current residential ZIP code. 

### 2.3. Neighborhood-Level Variables

Thirteen neighborhood-level socioeconomic status (SES) indicators were extracted from the ACS to develop an SES index of Florida neighborhoods (ZCTAs). These indicators were selected based on theoretical and empirical relevance, and covered a comprehensive range of domains, most commonly used in previous studies. Details are provided in a previous publication by Niyonsenga and the authors of the current study [30]. The thirteen indicators included were: percent of households without access to a car, percent of households with ≥1 person per room, percent of population living below the poverty line, percent of owner-occupied homes worth ≥$300,000, median household income in 2013, percent of households with annual income <$15,000, percent of households with annual income ≥$150,000, income disparity (derived from percent of households with annual income <$10,000 and percent of households with annual income ≥$50,000), percent of population aged ≥25 with less than a 12th grade education, percent of population aged ≥25 with a graduate professional degree, percent of households living in rented housing, percent of population aged ≥16 who were unemployed, and percent of population aged ≥16 employed in high working class occupation (ACS occupation group: “managerial, business, science, and arts occupations”). Income disparity was calculated as the logarithm of 100 times the percent of households with annual income <$10,000, divided by the percent of households with annual income ≥$50,000, and was used as a proxy for the Gini-coefficient [30]. All neighborhood-level indicators were coded so that higher scores corresponded to lower SES (higher disadvantage); they were then standardized.

To calculate the SES index, we started by conducting a reliability analysis. The Cronbach’s alpha for all 13 indicators was 0.93. We selected seven indicators based on the correlation of the indicator with the total index (high correlation), and the Cronbach’s alpha if the item was deleted (low alpha). The seven indicators selected were: percent below poverty, median household income, percent of households with annual income <$15,000, percent of households with annual income ≥$150,000, income disparity, percent of population age ≥25 with less than a 12th grade education, and high-class work. The resulting Cronbach’s alpha increased (0.94). 

Second, we conducted a principal component analysis with and without varimax rotation, which revealed one factor with an eigenvalue greater than one (5.14). This factor accounted for 73.49% of the variance in the indicators. Because all of the factor loadings were high (between 0.80 and 0.93), we retained all seven indicators. Finally, we added the standardized scores for the seven variables, and categorized these scores into quartiles. 

To categorize ZCTAs into rural or urban areas, we used Categorization C of Version 2.0 (University of Washington, Washington, DC, USA) of the Rural-Urban Commuting Area (RUCA) codes, developed by the University of Washington WWAMI Rural Research Center [31]. The percent of the area population who identified as Hispanic/Latino (Latino ethnic density), was extracted from the ACS and divided into three categories: <25%, 25–49%, and ≥50% [32,33].

### 2.4. Statistical Analyses

Individual- and neighborhood-level data were merged by matching the current ZIP code of each case, with the ZIP code’s corresponding ZCTA. First, we compared individual-level and neighborhood-level characteristics by country of birth (including race). Second, we compared outcomes by race and by country of birth by estimating crude and adjusted odds ratios, and 95% confidence intervals for non-retention in care during 2015 and non-viral suppression during 2015. Multi-level (Level 1: individual; Level 2: neighborhood) logistic regression modeling was used to account for correlation among cases living in the same neighborhood. To estimate the contribution of individual and neighborhood factors on racial/ethnic disparities, we first estimated crude ORs (Model 1), followed by ORs controlled for individual factors (Model 2), and ORs controlled for both individual and neighborhood factors (Model 3). Finally, to identify unique predictors of non–retention in care and non-viral suppression for each group, we estimated separate, fully adjusted models (Model 3), stratified by country of birth. Odds ratios were calculated for the year of HIV diagnosis, race, sex, age, mode of HIV transmission, AIDS diagnosis, neighborhood socioeconomic status (index of 7 indicators), rural/urban status, and percent Latino in the neighborhood. SAS software, version 9.4 (SAS Institute, Cary, NC 2002), was used to conduct analyses. The Florida International University Institutional Review Board approved this study (IRB-13-0193), and the Florida Department of Health Institutional Review Board designated this study as non-human subjects research.

## 3. Results

### 3.1. Characteristics of Participants

Of 18,793 Latinos diagnosed with HIV between 2000 and 2014 in Florida, 12,106 (64.4%) met our inclusion/exclusion criteria. Most individuals were of White race (93.9%, includes imputed data for race) (Table 1). U.S.-born Latinos accounted for the majority of cases (35.3%), followed by Latinos born in Cuba (21.6%). A larger proportion of individuals born in Cuba were 50 years of age or older (20.2%), when compared with individuals born in the U.S. (9.1%), Mexico (8.2%), and Central America (9.1%). A larger proportion of individuals born in Cuba (75.7%) and South American (71.2%) reported MSM as a potential mode of HIV transmission, when compared with fewer than 50% for those born in Mexico and Central America. Individuals born in South America were less likely to live in a neighborhood with low SES (26.6%), than those born in Central America (53.6%), Mexico (49.2%), and Cuba (46.4%). Finally, nearly 65% of those born in Cuba lived in a neighborhood in which the majority of the population was Latino. 

### 3.2. Retention in HIV Care

Overall, 31.9% of Latinos in our study were not retained in HIV care in 2015. The proportion of Latinos not retained in care by race, was similar (Black 33.9%, White 31.7%) (Table 2). Models adjusting for individual- and neighborhood-level factors showed similar non-significant results for non–retention in care by race. The proportion of Latinos who were born in Mexico (49.3%) and Central America (38.7%), who were not retained in care, was significantly higher than for Latinos born in the U.S. (32.7%). Differences remained significant after controlling for individual- and neighborhood-level factors (Mexico adjusted (aOR) 2.00, 95% confidence interval (CI) 1.70–2.36; Central America aOR 1.33, 95% CI 1.16–1.53). Within the Central America group, only those born in Guatemala (aOR 2.39, 95%CI 1.80–3.18) and Honduras (aOR 1.39, 95% CI 1.13–1.72) had significantly higher odds of non-retention in care, when compared with U.S.-born Latinos. More than 50% of Latinos born in Guatemala (53.2%) were not retained in HIV care. For both Mexican-born and Central American-born Latinos, being male was a risk factor (Mexico aOR 1.93, 95% CI 1.16–3.21; Central America aOR 2.47, 95% CI 1.73–3.55; not in table), as was not having AIDS (Mexico aOR 1.81, 95% CI 1.32–2.49; Central America 1.49, 95% CI 1.16–1.91, not in Table), for non-retention in care.

### 3.3. Viral Suppression

Overall, 35.9% of Latinos in our study were not virally suppressed in 2015. A larger proportion of Black Latinos (40.4%) were not virally suppressed, when compared with White Latinos (35.6%) (crude OR 1.23, 95% CI 1.05–1.43) (Table 3). The difference in the odds of non-viral suppression between Blacks and Whites remained after adjusting for individual–level variables, including country of birth (aOR 1.19, 95% CI 1.04–1.37), but not after controlling for both individual and neighborhood factors (aOR 1.16, 95% CI 0.98–1.36). Similar to retention in care, Latinos born in Mexico (52.7%) and Central America (43.3%) were more likely not to be virally suppressed when compared with U.S.-born Latinos (37.3%). These differences remained after controlling for individual and neighborhood factors (Mexico aOR 1.85, 95% CI 1.57–2.17; Central America aOR 1.28, 95% CI 1.12–1.47). Further examination of Central America showed that only Latinos born in Guatemala (aOR 2.20, 95% CI 1.66–2.92) and Honduras (aOR 1.42, 95% CI 1.16–1.74) had an elevated risk when compared with U.S.-born Latinos. Similar to non-retention in care, Latinos born in Mexico who were male, were at increased odds of non-viral suppression compared to their female counterparts (aOR 1.77, 95% CI 1.08–2.29), as were people who had not been diagnosed with AIDS (aOR 1.65, 95% CI 1.21–2.25) (not in Table). For Central American-born Latinos, being male (aOR 1.94, 95% CI 1.36–2.75), reporting injection drug use when compared with heterosexual contact (aOR 2.06, 95% CI 1.03–4.13), and not having AIDS (aOR 1.42, 95% CI 1.11–1.81), were all risk factors for non-viral suppression. The HIV transmission mode of MSM, when compared with heterosexual contact, was protective for those born in Central America (aOR 0.59, 95% CI 0.43–0.82). 

## 4. Discussion

Our study has three primary findings. First, our study suggests no significant racial differences in non-retention in HIV care between Black and White Latinos, and only marginal racial differences in non-viral suppression that appear to be explained by differences in individual and neighborhood characteristics. Second, Latinos born in Mexico and Central America were at increased odds of non-retention in care and non-viral suppression when compared with U.S.-born Latinos, even after controlling for individual- and neighborhood-level factors. Those born in Guatemala and Honduras were particularly at risk. Finally, among Mexican-born and Central American-born Latinos, being male nearly doubled the odds of non-retention in care and non-viral suppression. 

We did not find racial disparities in retention in HIV care among Latinos in our study. Although this is the first study to examine retention in HIV care among Latinos by race, we expected to find significant differences given the racial disparities in retention seen among non-Latinos [8,34,35,36,37]. Our findings are also inconsistent with reports suggesting that Black Latinos resemble non-Latino Blacks in access to care [38]. Furthermore, racial disparities among Latinos have been documented in delayed HIV diagnosis [15], and in non-HIV related outcomes, including birth outcomes [39]. Our study did show marginally significant disparities in viral load suppression, with Black Latinos exhibiting increased odds of non–viral suppression. This difference remained after controlling for year of diagnosis, sex, age, HIV transmission mode, and country of birth, suggesting that these individual-level variables did not explain the disparities. However, the effect of race on non-viral suppression disappeared after controlling for individual factors and neighborhood socioeconomic status, rural/urban residence, and Latino ethnic density. This may reflect the fact that Black Latinos experience higher poverty and unemployment, and lower educational attainment, when compared with White Latinos [17]. 

Latinos born in Mexico in our study had a two-fold increase in odds of non-retention in care and non-viral suppression, when compared with U.S.-born Latinos. Similar but less pronounced effects were seen among Latinos born in Central America. While these results are not consistent with a study of Latinos recruited in five Los Angeles HIV clinics [16], they are consistent with numerous studies reporting disparities in delayed HIV diagnoses [10,11,12], and survival [10,13,14,15] among these groups. It is worth noting that the study in Los Angeles had a relatively small sample of individuals born in Mexico (n = 109) and Central America (n = 36), and compared these groups with U.S.–born Latino and African American MSM and women [16]. Our study further identified Latinos born in Guatemala and Honduras as being at high risk. Also worth mentioning is the significant and protective effect of being born in Cuba, when compared with the U.S., for both retention in HIV care and viral suppression. These findings, and the fact that we did not find significant disparities by race, suggest that inequalities may be due to cultural differences, differences in health seeking behavior, and possible differences in immigration experience between Latinos born in Guatemala and Honduras, and U.S.–born Latinos. For example, a qualitative study of Central American- and Mexican-born Latinos living with HIV found that cultural factors, including gender roles and *machismo*, played a role in health care utilization. Individuals also reported a fear of being deported if they sought care [40]; a fear more likely applicable to Mexican- and Central American-born Latinos than Cuban-born Latinos due to differential U.S. immigration policies. Acculturation also plays a role in health-seeking behavior among Latinos. For example, a study of 219 Latinos with HIV (70% from Mexico and 24% from Central America), who were not fully engaged in HIV care or were at risk of falling out of care, found that less acculturated Latinos (whose primary language was Spanish) were more likely to experience stigma, belief, and structural barriers to care than their more acculturated English-speaking counterparts [41]. They also reported the tendency to seek care only when sick, because they are typically more concerned about making ends meet for their children. This could explain why not having AIDS was a risk factor for both retention in care and viral suppression. 

Finally, our study found males to be at increased odds of non-retention in care and non-viral suppression among Latinos born in Central America, Mexico, and South America, but not among U.S.-born Latinos. Although we were unable to run the full multilevel models on Latinos born in Guatemala and Honduras due to the smaller size of these groups, we were able to do some exploratory analyses. Models adjusting for individual–level factors only, suggested that males born in Guatemala were almost three times more likely not to be retained in care or be virally suppressed, when compared with their female counterparts (aOR 2.82, 95% CI 1.24–6.42; 2.82, 95% CI 1.23–6.42, respectively). Males born in Honduras were also at increased risk compared with females born in Honduras, for retention in care only (aOR 2.22, 95% CI 1.31–3.78). Our finding of lower retention in care and viral suppression among males is consistent with the literature [36,42,43,44,45], although a few studies have found males to be more likely to be virally suppressed [44], or have found no differences by gender [35]. It is possible that males born in these countries traveled to the U.S. alone, either as migrant workers, or to work permanently in the U.S. to provide for a family member in their home country. Participants of a qualitative study of HIV-infected Mexican, Central American, and Dominican immigrants, expressed that men who do not have families in the U.S. will not seek care because women typically make them go or increase the urgency of seeking care [40]. 

Our study has four main limitations. First, Latinos in our surveillance dataset who do not have evidence of being engaged in care may be getting care outside of the U.S. This data limitation is more likely among foreign-born Latinos. Related to this, our study assumes that Latinos not engaged in care are not virally suppressed. Second, data on race and country of birth was self-reported or abstracted from medical records. While country of birth is more objective, Latinos have reported being unclear or undefined about their racial category [46]. Third, we lacked data on ethnic origin for U.S.-born Latinos. Therefore, our discussion of ethnic and cultural differences between Latinos born in various countries/regions and U.S.-born Latinos is limited, and effect sizes seen in our study may be underestimated, depending on the ethnic origin distribution of our U.S.-born population. Additionally, the relatively small size of our population of Latinos born in Guatemala and Honduras prevented us from being able to conduct multilevel models to identify neighborhood-level risk factors for non-retention in care and non-viral suppression in these groups. Finally, our surveillance data lacked important information on individual-level socioeconomic status, years in the U.S. for foreign-born Latinos, health insurance status, and antiretroviral therapy. 

## 5. Conclusions

Our study identified significant disparities in retention in HIV care and viral load suppression among Mexican- and Central American-born Latinos. These disparities may explain the large differences in survival after HIV diagnosis among Latinos reported in numerous previous studies. The individual and neighborhood factors we examined in this study did not account for the disparities we found, and thus further studies may be needed to examine the mechanisms for these inequalities. Nevertheless, HIV clinics and providers must be aware of the increased risk of non-retention in care and non-viral suppression among these Latino groups when serving this community, and consider the implementation of culturally targeted health service strategies. Our study also suggests that male Latinos born in Mexico and Central America, particularly those born in Guatemala and Honduras, are at greatest risk. Decreasing barriers to care for Latino men, particularly those affecting men with low socioeconomic status, such as offering extended and weekend clinic hours, may need to be considered. Our study provides a clear rationale for studying Latinos by racial and country of birth subgroups, and it highlights the need for tailoring interventions to the unique healthcare and treatment barriers of each of these populations. 

## Figures and Tables

**Table 1:** Latinos age 13 and older diagnosed with HIV infection by birth country/region and selected characteristics, Florida, 2000–2014.

	All Latinos *^a^*	United States-Born Latinos	Puerto Rico	Mexico	Cuba	Central America *^b^*	South America *^c^*
Total (n)	12,106	4276	1153	842	2618	1284	1933
**Individual-Level Variables**							
Year of HIV diagnosis							
2000–2003	3007 (24.8)	954 (22.3)	341 (29.6)	254 (30.2)	527 (20.1)	309 (24.1)	622 (32.2)
2004–2007	3291 (27.2)	1204 (28.2)	286 (24.8)	266 (31.6)	605 (23.1)	408 (31.8)	522 (27.0)
2008–2011	3158 (26.1)	1233 (28.8)	290 (25.2)	189 (22.5)	679 (25.9)	354 (27.6)	413 (21.4)
2012–2014	2650 (21.9)	885 (20.7)	238 (20.5)	133 (15.8)	807 (30.8)	213 (16.6)	376 (19.5)
Race *^d^*							
Black	740 (6.1)	416 (9.7)	76 (6.6)	12 (1.4)	118 (4.5)	80 (6.1)	38 (2.0)
White	11,366 (93.9)	3860 (90.3)	1077 (93.4)	830 (98.6)	2500 (95.5)	1204 (93.8)	1895 (98.0)
Sex at birth							
Male	9997 (82.6)	3352 (78.4)	878 (76.2)	727 (86.3)	2388 (91.2)	983 (76.6)	1669 (86.3)
Female	2109 (17.4)	924 (21.6)	275 (23.9)	115 (13.7)	230 (8.8)	301 (23.4)	264 (13.7)
Age at diagnosis							
13–24	1630 (13.5)	865 (20.2)	125 (10.8)	125 (14.9)	205 (7.8)	157 (12.2)	153 (7.9)
25–49	8969 (74.1)	3023 (70.7)	840 (72.9)	648 (77.0)	1884 (72.0)	1010 (78.7)	1564 (80.9)
50 or older	1507 (12.5)	388 (9.1)	188 (16.3)	69 (8.2)	529 (20.2)	117 (9.1)	216 (11.2)
Mode of transmission							
IDU	860 (7.1)	392 (9.2)	233 (20.2)	41 (4.9)	89 (3.4)	44 (3.4)	61 (3.2)
MSM	7434 (61.4)	2506 (58.6)	526 (45.6)	413 (49.1)	1983 (75.7)	630 (49.1)	1376 (71.2)
Heterosexual	2901 (24.0)	1026 (24.0)	304 (26.4)	266 (31.6)	438 (16.7)	483 (37.6)	384 (19.9)
Other							
unknown	911 (7.5)	352 (8.2)	90 (7.8)	122 (14.5)	108 (4.1)	127 (9.9)	112 (5.8)
AIDS							
No	6906 (57.1)	2527 (59.1)	640 (55.5)	370 (43.9)	1633 (62.4)	586 (45.6)	1150 (59.5)
Yes	5200 (43.0)	1749 (40.9)	513 (44.5)	472 (56.1)	985 (37.6)	698 (54.4)	783 (40.5)
**ZCTA-Level Variables**							
SES index, quartiles							
1 (lowest)	4786 (39.5)	1532 (35.8)	424 (36.8)	414 (49.2)	1214 (46.4)	688 (53.6)	514 (26.6)
2	2825 (23.3)	1048 (24.5)	324 (28.1)	191 (22.7)	574 (21.9)	238 (18.5)	450 (23.3)
3	3001 (24.8)	1094 (25.6)	265 (23.0)	161 (19.1)	625 (23.9)	240 (18.7)	616 (31.9)
4 (highest)	1494 (12.3)	602 (14.1)	140 (12.1)	76 (9.0)	205 (7.8)	118 (9.2)	353 (18.3)
RUCA Classification							
Urban	11,870 (98.1)	4180 (97.8)	1123 (97.4)	785 (93.2)	2596 (99.2)	1279 (99.2)	1922 (99.4)
Rural	236 (2.0)	96 (2.3)	30 (2.6)	57 (6.8)	22 (0.8)	22 (0.8)	11 (0.6)
Percent of population Hispanic							
≥50	4694 (38.8)	1196 (28.0)	296 (25.7)	206 (24.5)	1697 (64.8)	562 (43.8)	737 (38.1)
25–49	3858 (31.9)	1429 (33.4)	402 (34.9)	274 (32.5)	640 (24.5)	382 (29.8)	731 (37.8)
<25	3554 (29.4)	1651 (38.6)	455 (39.5)	362 (43.0)	281 (10.7)	340 (26.5)	465 (24.1)

HIV, human immunodeficiency virus; IDU, injection drug use; MSM, male to male sexual contact; ZCTA, zip code tabulation area; SES, socioeconomic status. Percentage may not add up to 100 due to rounding. *^a^* Excludes cases diagnosed in a correctional facility (n = 322), missing current residential zip code (n = 2866) or had a reported zip code with an American Community Survey population of zero (n = 9), diagnosed under the age of 13 (n = 67), died before the year 2016 (n = 2751), and whose current state of residence was not Florida (n = 1077) or was missing (n = 407). Latinos born in an “unknown/unspecified” or missing country of birth (n = 1746) or in countries other than the U.S., Puerto Rico, Mexico, Cuba, Central America, and South America (n = 1001) were also excluded. *^b^* Includes Latinos born in Costa Rica (n = 56), El Salvador (n = 111), Guatemala (n = 245), Honduras (n = 496), Nicaragua (n = 299), and Panama (n = 77). *^c^* Includes Latinos born in Argentina (n = 278), Bolivia (n = 17), Chile (n = 78), Colombia (n = 672), Ecuador (n = 92), Paraguay (n = 18), Peru (n = 245), Uruguay (n = 50), and Venezuela (n = 483). *^d^* Race was missing for 988 Latinos that met our inclusion/exclusion criteria. After multiple imputation, 60 individuals were from a race other than Black or White (American Indian/Alaskan Native = 33, Asian = 14, Native Hawaiian/Pacific Islander = 13) and were excluded.

**Table 2:** Differences in non-retention in HIV care in 2015 by Black-White race and country of birth among Latinos aged 13 years and older diagnosed with HIV 2000–2014 in Florida.

			Model 1	Model 2	Model 3
All Latinos	Total, n	Not retained in care, n (%)	Crude OR for non-retention in care (95% CI)	Adjusted OR for non-retention in care (95% CI)	Adjusted OR for non-retention in care (95% CI)
Race					
Black	740	251 (33.9)	1.11 (0.94–1.29)	1.08 (0.92–1.28)	1.11 (0.93–1.31)
White	11,366	3605 (31.7)	Referent	Referent	Referent
Country of birth					
United States	4276	1399 (32.7)	Referent	Referent	Referent
Puerto Rico	1153	377 (32.7)	1.00 (0.87–1.15)	0.99 (0.85–1.14)	1.00 (0.86–1.16)
Mexico	842	415 (49.3)	2.00 (1.72–2.32)	1.99 (1.70–2.33)	2.00 (1.70–2.36)
Cuba	2618	493 (18.8)	0.48 (0.43–0.54)	0.54 (0.48–0.61)	0.52 (0.45–0.59)
Central America	1284	497 (38.7)	1.30 (1.14–1.48)	1.38 (1.20–1.58)	1.33 (1.16–1.53)
South America	1933	675 (34.9)	1.10 (0.99–1.24)	1.17 (1.04–1.32)	1.11 (0.98–1.26)
Central American and U.S. born	Total, n	Not retained in care, n (%)	Crude OR for non-retention in care (95% CI)	Adjusted OR for non-retention in care (95% CI)	Adjusted OR for non-retention in care (95% CI)
Country of birth					
United States	4276	1399 (32.7)	Referent	Referent	Referent
Costa Rica	56	22 (39.3)	1.33 (0.78–2.28)	1.37 (0.78–2.40)	1.36 (0.77–2.24)
Guatemala	248	132 (53.2)	2.34 (1.81–3.03)	2.44 (1.85–3.22)	2.39 (1.80–3.18)
Honduras	496	197 (39.7)	1.36 (1.12–1.64)	1.44 (1.18–1.76)	1.39 (1.13–1.72)
Nicaragua	299	90 (30.1)	0.89 (0.69–1.14)	0.99 (0.76–1.29)	0.94 (0.71–1.23)
Panama	77	21 (27.3)	0.77 (0.47–1.28)	0.89 (0.53–1.51)	0.90 (0.53–1.52)
El Salvador	111	38 (34.2)	1.07 (0.72–1.59)	1.03 (0.68–1.57)	1.03 (0.67–1.57)

OR, odds ratio; CI, confidence interval; Model 1: Crude rates; Model 2: Controlling for individual-level variables; Model 3: Controlling for individual-level variables and neighborhood-level variables.

**Table 3:** Differences in HIV non-viral load suppression (<200copies/mL) in 2015 by Black-White race and country of birth among Latinos aged 13 years and older diagnosed with HIV 2000–2014 in Florida.

			Model 1	Model 2	Model 3
All Latinos	Total, n	Not virally suppressed, n (%)	Crude OR for non-viral suppression (95% CI)	Adjusted OR for non-viral suppression (95% CI)	Adjusted OR for non-viral suppression (95% CI)
Race					
Black	740	299 (40.4)	1.23 (1.05–1.43)	1.19 (1.04–1.37)	1.16 (0.98–1.36)
White	11366	4049 (35.6)	Referent	Referent	Referent
Country of birth					
United States	4276	1595 (37.3)	Referent	Referent	Referent
Puerto Rico	1153	437 (37.9)	1.03 (0.90–1.17)	1.02 (0.89–1.17)	1.02 (0.89–1.18)
Mexico	842	444 (52.7)	1.88 (1.62–2.18)	1.87 (1.60–2.19)	1.85 (1.57–2.17)
Cuba	2618	595 (22.7)	0.50 (0.44–0.55)	0.58 (0.52–0.66)	0.56 (0.50–0.64)
Central America	1284	556 (43.3)	1.28 (1.13–1.46)	1.36 (1.19–1.55)	1.28 (1.12–1.47)
South America	1933	721 (37.3)	1.00 (0.90–1.12)	1.10 (0.98–1.24)	1.07 (0.95–1.21)
Central American and U.S. born	Total, n	Not virally suppressed, n (%)	Crude OR for non-viral suppression (95% CI)	Adjusted OR for non-viral suppression (95% CI)	Adjusted OR for non-viral suppression (95% CI)
Country of birth					
United States	4276	1595 (37.3)	Referent	Referent	Referent
Costa Rica	56	23 (41.1)	1.17 (0.69–2.00)	1.28 (0.74–2.22)	1.25 (0.71–2.18)
Guatemala	248	141 (56.9)	2.22 (1.71–2.89)	2.29 (1.74–3.07)	2.20 (1.66–2.92)
Honduras	496	227 (45.8)	1.41 (1.18–1.71)	1.52 (1.25–1.85)	1.42 (1.16–1.74)
Nicaragua	299	103 (34.5)	0.88 (0.69–1.13)	1.01 (0.78–1.30)	0.94 (0.73–1.23)
Panama	77	22 (28.6)	0.67 (0.41–1.11)	0.78 (0.47–1.30)	0.77 (0.46–1.29)
El Salvador	111	43 (38.7)	1.06 (0.72–1.57)	1.06 (0.71–1.58)	1.03 (0.68–1.54)

OR, odds ratio; CI, confidence interval; Model 1: Crude rates; Model 2: Controlling for individual-level variables; Model 3: Controlling for individual-level variables and neighborhood-level variables.
